# Short-Duration HIPEC-Mimetic Mithramycin A Exposure Induces Durable Transcriptional Remodeling Involving Chromatin Regulatory Networks in Colorectal Cancer Models

**DOI:** 10.3390/ijms27083580

**Published:** 2026-04-17

**Authors:** Olivia Coburn-Flynn, M. Virginia Butchy, Yazid Ghanem, Robert Emery, Vincent Verchio, Kristen Knapp, Jessica Collier, Sahil Jethi, Francis R. Spitz, Ping Zhang, Weam Othman Elbezanti, Young Ki Hong

**Affiliations:** 1 Department of Surgery, Cooper University Health Care, Camden, NJ 08103, USAzhang-ping@cooperhealth.edu (P.Z.); 2 Department of Biomedical Sciences, Cooper Medical School, Rowan University, Camden, NJ 08103, USA; 3 Department of Chemistry and Biochemistry, Rowan University, Glassboro, NJ 08028, USA; 4 Department of Surgery, Cooper Medical School, Rowan University, Camden, NJ 08103, USA; 5 Camden Cancer Research Center, Camden, NJ 08103, USA; 6 MD Anderson Cancer Center at Cooper, Camden, NJ 08103, USA

**Keywords:** mithramycin A, colorectal cancer, hyperthermic intraperitoneal chemotherapy, chromatin regulation, transcriptional remodeling, cell-cycle checkpoint, clonogenic survival, hyperthermia

## Abstract

Hyperthermic intraperitoneal chemotherapy (HIPEC) for colorectal peritoneal metastases relies primarily on DNA-damaging agents whose efficacy depends on sustained cytotoxic exposure. Whether brief treatment can induce durable transcriptional remodeling remains unclear. Mithramycin A (MA) is a GC-rich DNA-binding agent with transcriptional regulatory activity involving chromatin-associated pathways. Here, we investigated the molecular and functional consequences of a single 90-min HIPEC-mimetic MA exposure in colorectal cancer models. RNA sequencing revealed extensive and coordinated transcriptional remodeling, affecting a substantial fraction of expressed genes and producing a response qualitatively distinct from mitomycin C. MA selectively suppressed key chromatin-associated regulatory factors, including DNMT1, JARID2, and HDAC4, while coordinately activating canonical cyclin-dependent kinase inhibitors CDKN1A, CDKN1C, and CDKN2C. Gene set enrichment analysis demonstrated enrichment of G2/M checkpoint pathways and suppression of oncogenic gene networks. These molecular changes translated into sustained inhibition of clonogenic growth and activation of caspase-dependent apoptosis following drug washout, with hyperthermia potentiating apoptotic signaling. Collectively, these findings indicate that brief MA exposure induces selective modulation of chromatin regulators and durable transcriptional reorganization, supporting modulation of chromatin regulatory networks as a potential therapeutic strategy for HIPEC-based colorectal cancer therapy.

## 1. Introduction

Colorectal cancer (CRC) remains a leading cause of cancer-related mortality worldwide, and peritoneal metastasis represents a particularly challenging clinical scenario associated with poor prognosis and limited therapeutic options [1,2]. Hyperthermic intraperitoneal chemotherapy (HIPEC), administered in conjunction with cytoreductive surgery, has emerged as a locoregional treatment strategy to deliver high concentrations of chemotherapeutic agents directly to the peritoneal cavity while limiting systemic toxicity [3,4,5]. Despite its clinical utility in selected patients, the efficacy of HIPEC is largely dependent on DNA-damaging agents such as mitomycin C (MC) and oxaliplatin (OX), whose cytotoxic effects require sustained exposure and primarily rely on the induction of replication stress and genotoxic injury [6,7,8,9]. Resistance frequently develops, and durable disease control remains inconsistent, underscoring the need for mechanistically distinct therapeutic strategies.

Increasing evidence indicates that colorectal cancer progression and therapeutic resistance are driven not only by genetic mutations but also by epigenetic dysregulation and transcriptional plasticity [10,11,12]. Aberrant DNA methylation, histone modification, and chromatin remodeling contribute to the silencing of tumor suppressor genes, the maintenance of stemness-associated transcriptional programs, and adaptation to cytotoxic stress [13,14,15]. Key regulators of these processes—including DNA methyltransferase 1 (DNMT1), histone deacetylases (HDACs), and Polycomb-associated factors such as JARID2 and EZH2—have been implicated in colorectal tumorigenesis and treatment resistance [16,17,18,19,20,21,22,23,24,25]. These findings suggest that targeting epigenetic dependencies may represent a complementary or alternative strategy to conventional DNA-damaging chemotherapy.

Mithramycin A (MA) is a GC-rich DNA-binding transcriptional regulator originally developed as an antineoplastic antibiotic that inhibits transcription by interfering with SP1-dependent promoter activity [26,27,28,29]. By displacing SP1 and related transcription factors from GC-rich promoter regions, MA modulates the expression of multiple genes involved in oncogenic signaling and chromatin regulation [30,31,32]. Beyond its effects on individual oncogenes, MA has been shown to influence chromatin-associated pathways and alter the expression of chromatin-associated regulatory factors across multiple malignancies [31,33]. However, most prior studies have evaluated MA under prolonged exposure conditions, and its capacity to induce durable transcriptional remodeling following short-duration treatment—particularly under HIPEC-relevant hyperthermic conditions—remains insufficiently defined.

Given that HIPEC involves high local drug concentrations delivered over limited time intervals [34], we hypothesized that transient exposure to a transcriptional regulator capable of modulating chromatin regulatory networks could induce durable transcriptional remodeling distinct from the acute DNA damage response elicited by conventional HIPEC agents. Specifically, we postulated that short-duration MA exposure would selectively disrupt chromatin-associated regulatory pathways, reactivate tumor suppressor-associated transcriptional programs, and produce sustained growth inhibition following drug washout. To test this hypothesis, we integrated RNA sequencing, targeted qPCR validation, pathway-level analysis, and functional assays in colorectal cancer models under HIPEC-mimetic conditions. We demonstrate that transient MA treatment induces selective suppression of key chromatin regulatory factors, coordinated activation of canonical cyclin-dependent kinase inhibitors, structured reorganization of cancer-relevant transcriptional networks, and durable suppression of clonogenic growth. Collectively, these findings support a transcriptional remodeling-dominant mechanism that distinguishes MA from conventional HIPEC chemotherapeutics and position transcriptional state modulation as a potential strategy for locoregional colorectal cancer therapy.

## 2. Results

### 2.1. Short-Duration MA Exposure Induces Extensive and Structured Transcriptional Remodeling in HT-29 Cells

To determine whether brief exposure to MA is sufficient to induce durable transcriptional remodeling under HIPEC-mimetic conditions, we performed RNA sequencing in HT-29 colorectal cancer cells following a 90-min exposure and subsequent drug washout. MC, an FDA-approved HIPEC agent with DNA crosslinking activity, was evaluated in parallel to distinguish transcriptional remodeling from conventional genotoxic stress responses.

HT-29 cells were selected as the primary transcriptomic model due to their stemness-enriched and therapy-resistant phenotype, characterized by mutant TP53, active PI3K/AKT signaling, and reliance on transcriptional regulatory programs for survival [35,36]. In three-dimensional tumoroid systems, HT-29 cells exhibit elevated expression of stemness-associated markers, including LGR5 and CD133, and demonstrate transcriptional features linked to epithelial–mesenchymal transition and therapeutic resistance. Importantly, MA has previously demonstrated potent activity in HT-29 tumoroids, including suppression of SP1-dependent transcription and reduction of stemness markers. Collectively, these characteristics provide a biologically relevant and stringent context in which to assess whether short-duration MA exposure produces coordinated transcriptional reorganization rather than a transient stress-induced perturbation or nonspecific cytotoxic response. This model, therefore, enables interrogation of adaptive and resistance-associated transcriptional networks, rather than terminal cell death pathways alone.

**Figure 1:**
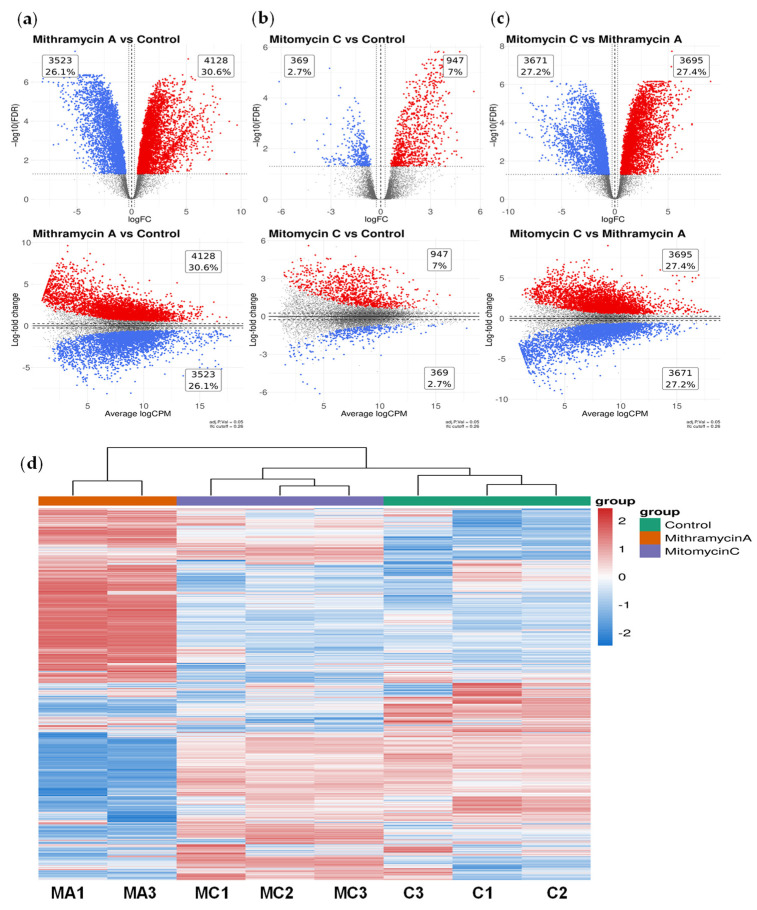
Short-duration Mithramycin A exposure induces extensive transcriptional remodeling in HT-29 colorectal cancer cells. (**a**) Volcano plot showing differential gene expression in HT-29 cells following a single 90-min exposure to Mithramycin A (MA) relative to untreated control. Significantly upregulated genes (red) and downregulated genes (blue) were defined as |log_2_ fold change| ≥ 1.5 with adjusted *p* < 0.05 (Benjamini–Hochberg correction). A total of 4128 genes were upregulated, and 3523 genes were downregulated. (**b**) Volcano plot showing differential gene expression following 90-min mitomycin C (MC) exposure under identical conditions relative to control. A total of 369 genes were upregulated, and 947 genes were downregulated (|log_2_ fold change| ≥ 1.5; adjusted *p* < 0.05). (**c**) Differential expression analysis directly comparing MA-treated and MC-treated samples, illustrating bidirectional transcriptional differences between treatments. (**d**) Unsupervised hierarchical clustering heatmap of significantly differentially expressed genes across control, MC-treated, and MA-treated samples following exclusion of the MA2 outlier sample (biological triplicates per condition), demonstrating segregation of treatment groups.

MA treatment resulted in extensive and coordinated transcriptional remodeling. Relative to untreated controls, 3523 genes (26.1%) were significantly downregulated, and 4128 genes (30.6%) were upregulated (FDR < 0.05; Figure 1a). In contrast, MC elicited a substantially more limited response, with 369 genes upregulated (2.7%) and 947 genes downregulated (7.0%) under identical exposure conditions (Figure 1b). The complete differential expression dataset, including all upregulated and downregulated genes, is provided in Supplementary Table S1.

Direct comparison between MA and MC demonstrated widespread bidirectional differences in gene expression (Figure 1c), indicating that MA induces a transcriptional response distinct from that observed with conventional chemotherapy. Principal component analysis (PCA) demonstrated clear segregation of MA-treated samples from both untreated and MC-treated groups, while one MA replicate (MA2) exhibited separation from the remaining MA samples (Supplementary Figure S4). Consistent with this observation, quality control metrics revealed elevated duplication rates and reduced mapping efficiency in the MA2 sample relative to other replicates (Supplementary Figure S5). Based on these standard quality-control criteria, MA2 was excluded from downstream analyses. Importantly, transcriptional patterns remained highly consistent when analyses were performed, including or excluding this sample (Supplementary Figure S3).

Unsupervised hierarchical clustering of the noOutlier dataset further demonstrated clear segregation of MA-treated samples from both untreated and MC-treated groups (Figure 1d), consistent with induction of a discrete and structured transcriptional state following transient MA exposure. A corresponding heatmap generated from the full dataset, including MA2, is provided in Supplementary Figure S1 and supports the overall robustness of the transcriptional signature.

Collectively, these findings demonstrate that short-duration MA treatment is sufficient to trigger large-scale and organized transcriptional remodeling in a therapy-resistant colorectal cancer model, consistent with a structured transcriptional response distinct from DNA damage-based chemotherapy.

### 2.2. Validation of Chromatin Regulatory Targets Confirms Selective On-Target MA Activity

Given the scale and structured nature of MA-induced transcriptional remodeling, we next examined whether these changes reflected engagement of established chromatin-associated regulatory mechanisms implicated in SP1-dependent transcriptional control.

**Figure 2:**
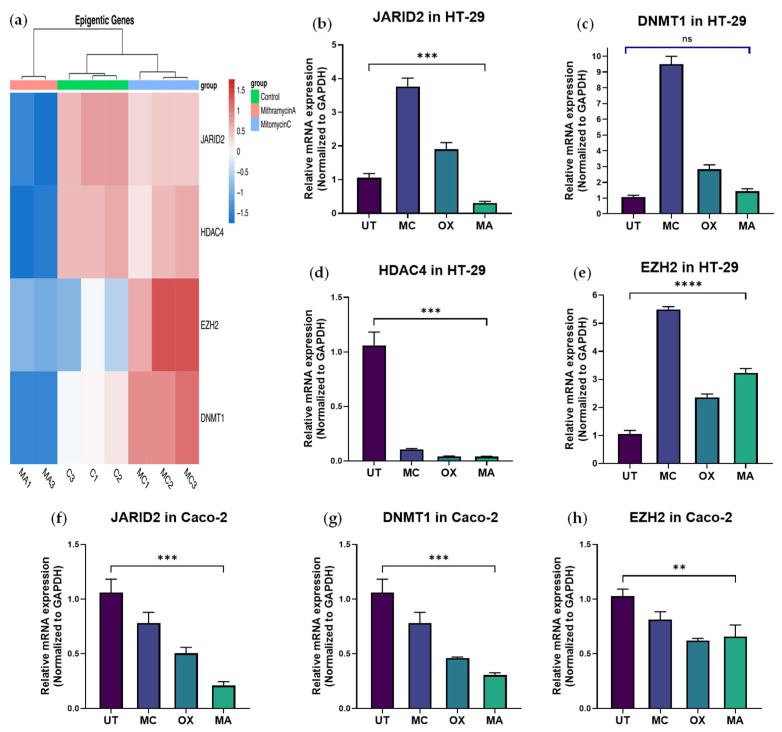
Validation of chromatin regulatory factor modulation by Mithramycin A under HIPEC-mimetic conditions. Quantitative PCR analysis of chromatin-associated regulatory factors in colorectal cancer cell lines following 90-min exposure to Mithramycin A (MA), mitomycin C (MC), or oxaliplatin (OX), followed by drug washout and incubation in fresh medium for 48 h. (**a**) Heatmap representation of relative mRNA expression of chromatin regulatory genes (DNMT1, JARID2, EZH2, and HDAC4) across treatment groups in HT-29 and Caco-2 cells, illustrating selective modulation by MA compared with MC and OX. ((**b**–**e**) Relative mRNA expression of JARID2 (**b**), DNMT1 (**c**), HDAC4 (**d**), and EZH2 (**e**) in HT-29 cells. (**f**–**h**) Relative mRNA expression of JARID2 (**f**), DNMT1 (**g**), and EZH2 (**h**) in Caco-2 cells. Gene expression levels were normalized to GAPDH and calculated using the 2^−ΔΔCt method relative to untreated controls (set to 1). Data are presented as mean ± SD (or SEM—match your analysis) from at least three independent experiments. Statistical comparisons were performed between MA and UT conditions using a two-tailed unpaired Student’s *t*-test. ns, not significant ** *p* < 0.01; *** *p* < 0.001; **** *p* < 0.0001.

RNA sequencing analysis of annotated chromatin-modifying enzymes revealed selective modulation of chromatin-associated regulatory factors following MA exposure. While the majority of chromatin regulators did not demonstrate significant expression changes, a defined subset was consistently downregulated. Among these, DNMT1, JARID2, and HDAC4 exhibited significant suppression (Figure 2a). DNMT1 maintains DNA methylation patterns associated with oncogenic gene silencing [14], JARID2 functions as a regulatory component of the PRC2/EZH2 complex governing histone methylation [18], and HDAC4 contributes to transcriptional repression and cancer progression [17].

Quantitative PCR under HIPEC-mimetic conditions confirmed selective modulation of chromatin regulatory targets. In HT-29 cells, MA significantly reduced JARID2 and HDAC4 expression relative to untreated controls (Figure 2b,d), whereas conventional cytotoxic agents produced minimal or inconsistent effects. These findings validate the on-target modulation of specific chromatin regulators by MA.

In contrast, DNMT1 expression in HT-29 did not exhibit sustained suppression at 48 h after drug washout (Figure 2c), and EZH2 levels demonstrated a modest increase relative to control (Figure 2e). The divergence between early RNA-seq findings and later qPCR measurements likely reflects temporal dynamics of transcriptional regulation. RNA sequencing captures acute transcriptional responses immediately following MA exposure, whereas assessment at 48 h after drug washout evaluates stabilization of the reprogrammed transcriptional state. The absence of sustained DNMT1 suppression and the modest induction of EZH2 suggest selective and time-dependent modulation of chromatin regulatory networks rather than uniform or irreversible repression of chromatin regulatory factors.

In Caco-2 cells, MA similarly reduced JARID2 and DNMT1 expression (Figure 2f,g), whereas EZH2 was significantly suppressed (Figure 2h), indicating cell-line–dependent chromatin-associated transcriptional responses.

Collectively, these data demonstrate that MA selectively perturbs specific chromatin regulatory nodes—most consistently JARID2—while exerting context- and time-dependent effects on DNMT1, HDAC4, and EZH2. Importantly, MA does not function as a uniform inhibitor of individual epigenetic enzymes but rather modulates transcriptional networks through disruption of SP1-dependent regulatory circuitry. The observed cell line–specific and temporal variability is therefore consistent with differential chromatin states and adaptive regulatory responses across colorectal cancer models, rather than reflecting nonspecific or unstable gene regulation.

### 2.3. Identification of MA-Regulated Cell-Cycle Control Genes

Having established selective modulation of chromatin regulatory factors, we next sought to define coordinated transcriptional programs associated with sustained growth restraint.

Using predefined statistical thresholds (|log_2_FC| ≥ 1.5; adjusted *p* < 0.05), RNA sequencing identified 4128 upregulated and 3523 downregulated genes following MA exposure. To prioritize clinically relevant candidates, differentially expressed genes were intersected with curated oncologic databases, including the COSMIC Cancer Gene Census (733 genes) and the colorectal cancer Tumor Suppressor Gene Database (535 genes), yielding 326 cancer-associated MA-responsive genes.

This cancer-enriched subset was interrogated using gene ontology analysis focused on cell-cycle regulation, checkpoint signaling, and negative regulation of proliferation. In parallel, Gene Set Enrichment Analysis (GSEA) demonstrated enrichment of the Hallmark G2/M checkpoint pathway and tumor suppressor-associated signatures, alongside suppression of oncogene-associated gene sets.

**Figure 3:**
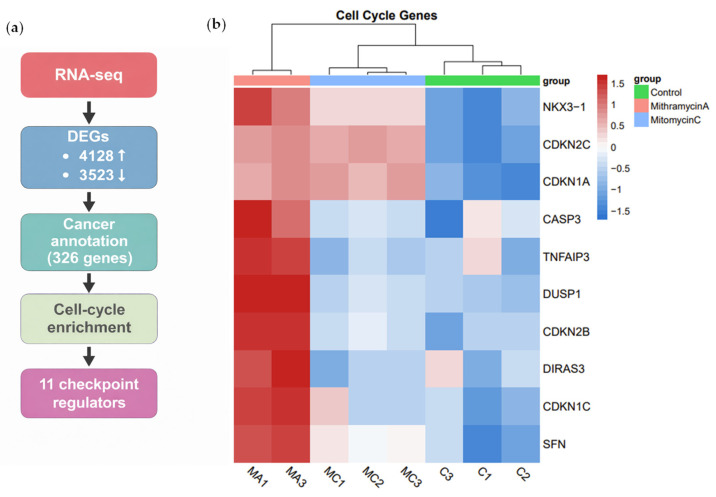
Workflow for identification of MA-selective cell-cycle regulatory genes. (**a**) Schematic overview of the analytic pipeline used to identify cancer-relevant cell-cycle genes selectively regulated by short-duration Mithramycin A (MA) exposure. RNA sequencing of HT-29 cells following a single 90-min treatment identified 4128 upregulated and 3523 downregulated genes (|log_2_FC| ≥ 1.5; FDR < 0.05). Differentially expressed genes were intersected with curated cancer gene databases (COSMIC Cancer Gene Census and colorectal cancer tumor suppressor gene sets), yielding 326 cancer-associated MA-responsive genes. Subsequent gene ontology filtering for cell-cycle–related biological processes identified 11 candidate checkpoint regulators. (**b**) Representative MA-responsive cell-cycle genes identified through this workflow.

Layered filtering identified 11 unique MA-responsive genes implicated in checkpoint enforcement (Figure 3a,b). Among these, canonical cyclin-dependent kinase inhibitors CDKN1A (p21), CDKN1C (p57), and CDKN2C (p18) were significantly upregulated, along with proliferation-restricting genes including DUSP1 and DIRAS3.

Importantly, several of these genes showed minimal or inconsistent regulation following MC treatment, indicating selective activation by MA rather than generalized chemotherapy-induced stress. Collectively, these findings demonstrate that transient MA exposure preferentially engages tumor-suppressive and checkpoint-enforcing transcriptional circuitry, reinforcing a transcriptional remodeling-dominant mechanism distinct from DNA damage-based chemotherapy.

### 2.4. Coordinated Induction of Canonical CDK Inhibitors Across Colorectal Cancer Models

To validate the functional relevance of checkpoint genes identified through transcriptomic filtering and to assess reproducibility across genetically distinct colorectal cancer models, quantitative PCR was performed in HT-29 and Caco-2 cells following 90-min drug exposure and washout.

**Figure 4:**
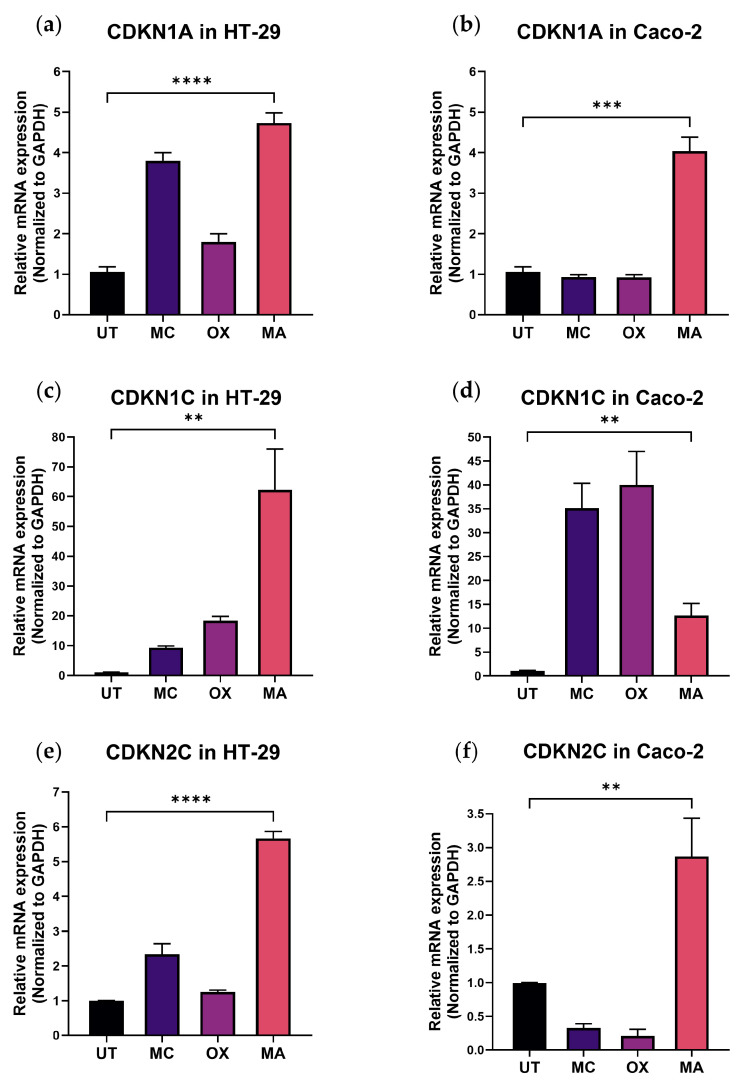
MA induces coordinated upregulation of canonical cyclin-dependent kinase inhibitors in colorectal cancer cells. Quantitative PCR analysis of CDKN1A, CDKN1C, and CDKN2C expression in HT-29 and Caco-2 colorectal cancer cells following 90-min treatment with vehicle control (UT), Mitomycin C (MC), Oxaliplatin (OX), or Mithramycin A (MA). (**a**,**c**,**e**) Relative mRNA expression in HT-29 cells. (**b**,**d**,**f**) Relative mRNA expression in Caco-2 cells. Data are presented as mean ± SD from three independent experiments performed in triplicate. Statistical comparisons were performed between MA and UT conditions using a two-tailed unpaired Student’s *t*-test. ** *p* < 0.01; *** *p* < 0.001; **** *p* < 0.0001.

MA consistently induced upregulation of canonical cyclin-dependent kinase inhibitors across both models. In HT-29 cells, CDKN1A and CDKN2C increased approximately 4–5-fold relative to control (Figure 4a,e), whereas CDKN1C exhibited markedly stronger induction (~60–70-fold; Figure 4c). In Caco-2 cells, MA induced CDKN1A (~3–4-fold; Figure 4b), CDKN1C (~10-fold; Figure 4d), and CDKN2C (~2–3-fold; Figure 4f).

In contrast, conventional chemotherapy agents produced more modest or variable transcriptional responses across cell lines. Although additional MA-responsive genes demonstrated cell-line–dependent variability, induction of CDKN1A, CDKN1C, and CDKN2C was reproducible in both models, supporting their consistent association with MA-driven growth restraint.

The magnitude and cross-model consistency of CDK inhibitor induction indicate that transient MA exposure activates a structured and conserved transcriptional program centered on checkpoint activation and proliferation control.

### 2.5. Gene Set Enrichment Analysis Reveals Structured Transcriptional Reorganization Following MA Exposure

To determine whether MA-regulated genes exhibited coordinated pathway-level organization, Gene Set Enrichment Analysis (GSEA) was performed using ranked RNA-seq data.

**Figure 5:**
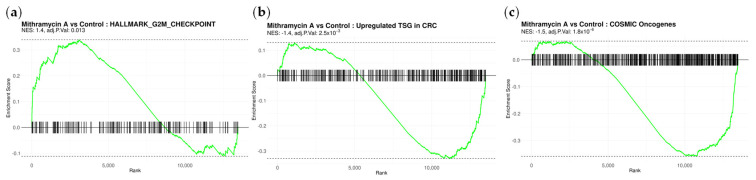
Gene Set Enrichment Analysis reveals structured transcriptional remodeling following MA exposure in colorectal cancer cells. (**a**) GSEA of RNA-seq data ranked by differential expression following Mithramycin A (MA) treatment demonstrates significant enrichment of the Hallmark G2M checkpoint pathway (NES = 1.42; nominal p = 0.0029; FDR q = 0.0134), indicative of transcriptional activation of cell-cycle control programs. (**b**) MA treatment is associated with significant suppression of oncogenic gene sets defined by the COSMIC database, reflecting downregulation of tumor-promoting transcriptional networks. (**c**) Concurrently, MA induces enrichment of colorectal cancer tumor suppressor gene sets among upregulated transcripts, demonstrating selective activation of antitumor pathways.

MA treatment was associated with significant enrichment of the Hallmark G2/M checkpoint pathway (NES = 1.42; FDR q = 0.013; Figure 5a), consistent with enrichment of transcriptional programs governing cell-cycle control. Correlation analysis demonstrated high concordance between full and noOutlier datasets at the pathway level (Supplementary Figure S3), supporting robustness of enrichment results to outlier exclusion. Concurrently, MA exposure resulted in significant suppression of COSMIC-defined oncogene gene sets (Figure 5b) and enrichment of colorectal cancer tumor suppressor gene sets among upregulated transcripts (Figure 5c). These enrichment patterns demonstrate that MA-induced transcriptional changes are highly structured and selectively target cancer-relevant regulatory networks.

Importantly, pathway enrichment analysis was used to contextualize gene-level validation findings rather than serve as standalone evidence of functional activation. Enrichment of checkpoint pathways aligned with the robust induction of CDKN1A, CDKN1C, and CDKN2C observed by qPCR, reinforcing the conclusion that MA engages coordinated growth-inhibitory transcriptional circuitry.

Additional gene ontology analysis of MA-downregulated transcripts revealed enrichment of pathways related to ribosomal biogenesis, small GTPase signaling, and cell junction organization (Supplementary Figure S2), suggesting broader remodeling of proliferative and structural networks.

Taken together, this integrated analysis—spanning cancer gene prioritization, targeted validation, and pathway-level enrichment—demonstrates that brief MA exposure initiates a durable and structured transcriptional state mechanistically distinct from DNA damage-based chemotherapy. Rather than inducing indiscriminate transcriptional suppression, MA selectively disrupts oncogenic regulatory networks while activating tumor suppressive and checkpoint-enforcing programs, providing a molecular framework for the sustained antiproliferative effects observed following treatment.

### 2.6. Transient MA Exposure Produces Durable Suppression of Clonogenic Growth

To determine whether the structured transcriptional remodeling induced by brief MA exposure translated into sustained biological consequences under HIPEC-relevant conditions, clonogenic survival assays were performed following short-duration treatment at normothermic (37 °C) and hyperthermic (42 °C) temperatures and subsequent drug washout. Following transient drug exposure and washout, cells were maintained under clonogenic conditions for 14 days in drug-free growth medium prior to fixation and colony quantification. This assay was specifically selected to evaluate long-term proliferative capacity rather than acute cytotoxicity, thereby assessing the durability of MA-induced transcriptional state remodeling.

**Figure 6:**
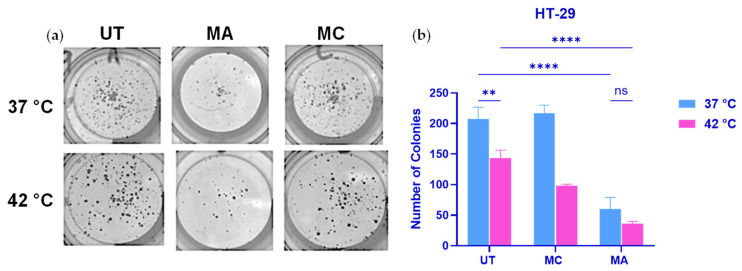
Transient MA exposure durably suppresses clonogenic growth of HT-29 colorectal cancer cells compared to MC. (**a**) Representative images of crystal violet-stained colonies formed by HT-29 cells following 90-min exposure to untreated control (UT), MA, or MC under normothermic (37 °C) or hyperthermic (42 °C) conditions. After drug washout, cells were cultured under clonogenic conditions for 14 days prior to fixation and staining with 0.5% crystal violet. (**b**) Quantification of clonogenic survival, expressed as the total number of colonies per well and normalized to untreated controls. Data represent mean ± SD from at least three independent experiments. Statistical analysis was performed using two-way ANOVA with Šídák’s post hoc test. ns, not significant; ** *p* < 0.01; **** *p* < 0.0001.

Quantitative analysis demonstrated that MA markedly reduced clonogenic growth relative to both untreated controls and MC-treated cells. Under normothermic conditions (37 °C), MA treatment resulted in an approximately 71% reduction in colony formation compared with the control, corresponding to an estimated 3.4-fold decrease in clonogenic survival (Figure 6). In contrast, MC did not significantly alter colony formation at 37 °C.

Under hyperthermic conditions (42 °C), MA reduced colony formation by approximately 74% relative to untreated controls. Notably, the magnitude of MA-mediated suppression was comparable at 37 °C and 42 °C. Thus, hyperthermia did not significantly enhance MA-mediated suppression of colony formation under the conditions tested. In contrast, MC demonstrated enhanced activity at 42 °C, producing an approximately 31% reduction in colony number compared with the control, whereas it had minimal effect at 37 °C.

Importantly, colonies were assessed after 14 days in drug-free conditions, indicating that these effects reflect persistent impairment of proliferative capacity rather than transient growth delay. Collectively, these findings demonstrate that brief MA exposure induces durable suppression of clonogenic potential independent of temperature, whereas MC exhibits partial temperature-dependent enhancement. These results highlight mechanistic divergence between transcriptional remodeling-driven growth restraint and hyperthermia-potentiated DNA crosslink-mediated cytotoxicity.

### 2.7. Transient MA Exposure Engages Caspase-Dependent Apoptotic Programs Potentiated by Hyperthermia

To assess cell fate programs associated with transient MA exposure, we interrogated activation of caspase-dependent apoptotic signaling at both transcript and protein levels. Within a HIPEC-mimetic framework, cells were exposed to Mithramycin A (MA) for 90 min under normothermic (37 °C) or mild hyperthermic (42 °C) conditions, followed by drug washout to model clinically relevant transient intraperitoneal chemotherapy exposure. Apoptotic signaling was assessed 48 h post-treatment to determine whether brief MA exposure—alone or combined with hyperthermia—drives durable commitment to cell death.

**Figure 7:**
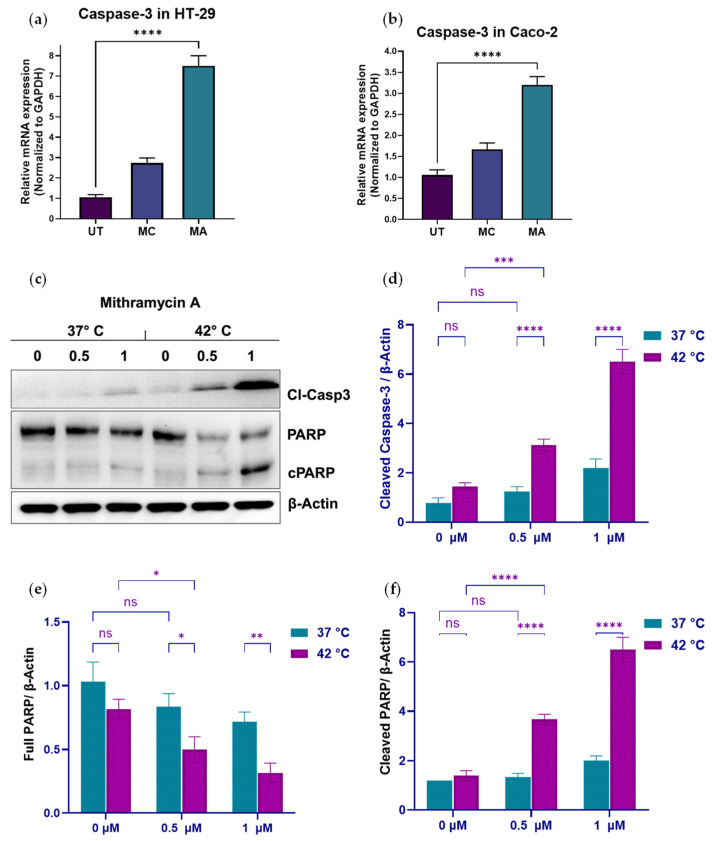
MA activates caspase-dependent apoptosis potentiated by hyperthermia. (**a**,**b**) Quantitative PCR analysis of CASP3 mRNA expression in HT-29 (**a**) and Caco-2 (**b**) cells following 90-min exposure to mithramycin A (MA) or mitomycin C (MC), followed by drug washout. Bars represent treatment groups: UT (untreated), MC (mitomycin C), and MA (mithramycin A). Expression levels were normalized to GAPDH and are presented relative to untreated controls (UT). Data represent mean ± SD from three independent experiments. (**c**) Representative Western blot analysis of cleaved caspase-3 (Cl-Casp3), cleaved PARP (cPARP), full-length PARP, and β-actin in cells treated with increasing concentrations of MA (0, 0.5, and 1 µM) under normothermic (37 °C) or mild hyperthermic (42 °C) conditions for 90 min, followed by washout and analysis 48 h post-treatment. (**d**) Densitometric quantification of cleaved caspase-3 normalized to β-actin. (**e**) Quantification of full-length PARP normalized to β-actin. (**f**) Quantification of cleaved PARP normalized to β-actin. Data in (**d**–**f**) represent mean ± SD from three independent experiments. Statistical analysis was performed using two-way ANOVA with Šidák’s multiple comparisons test. ns, not significant; * *p* < 0.05; ** *p* < 0.01; *** *p* < 0.001; **** *p* < 0.0001.

Quantitative PCR demonstrated significant induction of *CASP3* transcripts following MA exposure. In HT-29 cells at 37 °C, MA increased *CASP3* expression approximately 7–8-fold relative to control, whereas MC produced a more modest ~3-fold induction (Figure 7a). A similar pattern was observed in Caco-2 cells, in which MA treatment resulted in an approximately 3-fold increase relative to untreated control.

Consistent with transcriptional activation, immunoblot analysis revealed dose-dependent increases in cleaved caspase-3 (Cl-Casp3) and cleaved PARP (cPARP) following MA exposure. At 37 °C, 1 µM MA increased Cl-Casp3 and cPARP levels approximately 2–3-fold relative to untreated control. Concurrent exposure to 42 °C and 1 µM MA resulted in markedly enhanced activation, with Cl-Casp3 and cPARP levels elevated approximately 4.5–5-fold. Correspondingly, full-length PARP levels decreased, consistent with increased proteolytic cleavage.

Quantification of the apoptotic index—calculated as the ratio of cleaved PARP to full-length PARP normalized to β-actin—demonstrated significant potentiation by hyperthermia. At 37 °C, 1 µM MA induced an approximately 2.9-fold increase in apoptotic index relative to control, whereas combined treatment with 42 °C and 1 µM MA produced a nearly 13-fold increase compared with untreated normothermic cells.

Collectively, these findings demonstrate that transient MA exposure is sufficient to engage caspase-dependent apoptotic programs and that mild hyperthermia substantially lowers the threshold for apoptotic commitment. Importantly, apoptotic activation occurs in the context of previously observed modulation of chromatin regulators, checkpoint-associated gene induction, and durable clonogenic suppression. Together, these data are consistent with a model in which MA-induced transcriptional alterations may contribute to increased susceptibility to apoptotic engagement, rather than reflecting solely acute cytotoxic stress.

## 3. Discussion

This study demonstrates that short-duration, HIPEC-mimetic exposure to MA induces extensive and structured transcriptional remodeling in colorectal cancer models that is mechanistically distinct from the DNA damage-dominated response elicited by conventional HIPEC agents [37]. Whereas MC and OX primarily exert cytotoxicity through DNA crosslinking, replication stress, and checkpoint collapse [6,7,8,9], transient MA exposure produced selective modulation of chromatin-associated regulatory factors, coordinated activation of canonical cell-cycle inhibitors, pathway-level reorganization of oncogenic transcriptional networks, and durable impairment of clonogenic survival following drug washout. Collectively, these findings indicate that brief transcriptional disruption under HIPEC-mimetic conditions is associated with sustained transcriptional and functional alterations in colorectal cancer cells.

Accumulating evidence indicates that colorectal cancer progression and therapeutic resistance are sustained not only by genomic alterations but also by epigenetic plasticity and transcriptional adaptability [10,14,15]. Maintenance of DNA methylation by DNMT1, histone deacetylation mediated by HDAC family members, and Polycomb-associated repression involving components such as JARID2 and EZH2 contribute to stemness maintenance, stress tolerance, epithelial–mesenchymal transition, and chemotherapy resistance [11,12,17]. Chromatin-associated regulatory reconfiguration has further been implicated in tumor recurrence, metastatic colonization, and adaptive resistance to cytotoxic therapies [14,38,39]. In this context, therapeutic strategies that destabilize chromatin regulatory maintenance networks may impose longer-lasting growth constraints than agents that rely exclusively on DNA damage induction.

Within this framework, the selective suppression of JARID2 and HDAC4, together with context-dependent modulation of DNMT1 and EZH2 following transient MA exposure, suggests targeted perturbation of chromatin regulatory nodes that sustain malignant transcriptional programs. Importantly, these effects were observed after brief exposure and remained detectable at later timepoints following drug washout, indicating that MA does not merely induce acute transcriptional repression but instead initiates regulatory adjustments that extend beyond the immediate treatment window. The divergence between acute RNA-seq findings and later qPCR measurements further underscores the temporal dynamics of chromatin-associated transcriptional remodeling, consistent with selective stabilization of specific regulatory alterations rather than uniform or irreversible chromatin suppression. The cell-line–dependent regulation of EZH2 indicates that MA does not induce indiscriminate Polycomb inhibition but instead modulates distinct chromatin regulatory dependencies according to cellular context, reflecting differences in baseline chromatin architecture across colorectal cancer subtypes.

This selective and temporally dynamic pattern aligns with prior studies demonstrating that MA disrupts SP1-dependent transcription and binds GC-rich promoter regions [40,41,42], thereby influencing multiple oncogenic and chromatin-associated regulatory programs simultaneously. Because SP1 regulates an extensive network of proliferation-, survival-, and chromatin-associated genes, transient interference with SP1-driven transcription may destabilize higher-order regulatory architecture rather than single downstream effectors. Consistent with the durable impairment of clonogenic survival observed following washout, our findings support a model in which short-duration SP1-targeted therapy induces coordinated transcriptional recalibration in colorectal cancer cells.

At the transcriptome-wide level, MA induced widespread yet structured remodeling, with enrichment of G2/M checkpoint pathways and tumor suppressor-associated gene sets, alongside suppression of oncogene-associated signatures. These pathway-level shifts demonstrated coordinated enrichment patterns involving transcriptional programs governing cell-cycle progression and proliferative signaling. Enrichment of checkpoint-associated pathways was consistent with gene-level validation findings, supporting the internal coherence of the observed transcriptional response.

The coordinated induction of CDKN1A, CDKN1C, and CDKN2C, together with enrichment of G2/M checkpoint pathways, supports engagement of structured cell-cycle regulatory mechanisms [43]. These transcriptional findings, together with the observed antiproliferative and pro-apoptotic effects, are consistent with functional engagement of cell-cycle arrest mechanisms.

CDKN1A (p21) and CDKN1C (p57) function as canonical cell-cycle checkpoint regulators and are frequently dysregulated or epigenetically suppressed in colorectal cancer, where reduced expression has been associated with enhanced proliferative capacity and adverse clinical features [43,44,45]. Restoration of their expression following brief MA exposure is therefore consistent with reactivation of endogenous checkpoint enforcement mechanisms rather than nonspecific cytotoxic injury.

The pronounced induction of CDKN1C in HT-29 cells is notable, given its established tumor-suppressor role and frequent epigenetic silencing in advanced malignancies. The magnitude of CDKN1C upregulation is consistent with a potential contributory role in MA-associated growth restraint, particularly within transcriptionally plastic, stemness-enriched colorectal cancer contexts. Importantly, although conventional chemotherapy can activate CDK inhibitors as part of a DNA damage response, the coordinated induction observed here—together with structured oncogene suppression and chromatin regulator modulation—is more consistent with a structured transcriptional response than with a purely generalized stress program. Future studies incorporating direct cell-cycle analyses will be important to further validate the functional consequences of checkpoint pathway activation observed in this study.

Consistent with these molecular findings, MA produced durable suppression of clonogenic survival following washout, indicating that transient transcriptional disruption translated into sustained functional impairment. The magnitude of colony formation reduction exceeded that observed with MC under identical conditions, supporting the interpretation that MA engages growth-inhibitory mechanisms distinct from those associated with DNA crosslink-mediated cytotoxicity. Notably, whereas MC demonstrated partial temperature-dependent enhancement, MA-mediated clonogenic suppression was robust and comparable under both normothermic and hyperthermic conditions. Together, these findings indicate that MA induces durable proliferative constraints that are preserved under hyperthermic conditions, while hyperthermia selectively potentiates apoptotic commitment rather than further suppressing clonogenic survival.

A defining feature of HIPEC is limited drug exposure duration. Agents whose therapeutic activity depends primarily on acute cytotoxicity during this exposure window may demonstrate limited persistence of effect following drug removal. In contrast, clonogenic assays demonstrated that a single 90-min MA treatment resulted in sustained suppression of proliferative capacity, as evidenced by markedly reduced colony formation after 14 days of drug-free growth. The durability of this effect is consistent with models of transcriptional dependency, in which cancer cells rely on sustained activity of specific transcriptional programs to maintain malignant phenotypes [46,47,48]. Transient disruption of such regulatory networks may therefore induce prolonged functional consequences that extend beyond the period of drug exposure. Previous studies demonstrate that MA suppresses SP1-dependent oncogenic drivers, including MYC and β-catenin-associated transcriptional targets, and inhibits tumor growth in preclinical models [40]. These findings support its characterization as a transcriptional network modulator rather than a conventional cytotoxic agent.

Within the HIPEC-mimetic framework, mild hyperthermia further potentiated caspase-dependent apoptosis. Hyperthermia has been reported to sensitize tumor cells to stress-induced signaling pathways and enhance treatment responsiveness [46,47]. The observed enhancement of apoptotic commitment under hyperthermic conditions suggests that temperature may amplify vulnerabilities created by MA-mediated disruption of chromatin regulatory networks. Importantly, apoptosis occurred in the context of prior checkpoint activation and durable clonogenic suppression, supporting a model in which transcriptional state remodeling lowers the threshold for apoptotic engagement rather than acting solely through acute cytotoxic mechanisms.

Given the hyperthermic conditions used in this model, it is important to consider whether the observed transcriptional changes may reflect general stress-response pathways. Although hyperthermic exposure can activate general cellular stress-response pathways, the transcriptional program observed following MA treatment was characterized predominantly by enrichment of cell-cycle checkpoint pathways and tumor suppressor-associated gene sets rather than canonical heat-shock responses. The coordinated induction of CDKN1A, CDKN1C, and CDKN2C, together with enrichment of G2/M checkpoint pathways, supports engagement of structured cell-cycle regulatory mechanisms. While a contribution from general stress-response pathways cannot be excluded, the overall transcriptional profile was not dominated by classical heat-shock genes, suggesting that the observed transcriptional remodeling reflects treatment-specific regulatory effects.

Collectively, these findings suggest a complementary therapeutic approach for locoregional colorectal cancer treatment. Traditional HIPEC agents rely predominantly on DNA crosslinking and replication stress-mediated cytotoxicity [6,7,8,9], yet resistance frequently emerges through enhanced DNA repair mechanisms and epigenetically mediated transcriptional plasticity [7,8,12,48]. Our data indicate that transient exposure to a transcriptionally active compound can induce coordinated reorganization of oncogenic gene expression programs and promote checkpoint-associated signaling. If validated in vivo, such a response may support HIPEC-based strategies that incorporate transcriptional state modulation alongside conventional genotoxic mechanisms.

Future investigations should extend these findings to in vivo models of peritoneal metastasis and compare transcriptomic signatures across multiple HIPEC-relevant agents to determine whether MA induces a uniquely structured transcriptional remodeling profile. Temperature-dependent transcriptomic and epigenomic profiling would clarify the mechanistic contribution of hyperthermia. Integration of chromatin accessibility (ATAC-seq), histone modification mapping, and DNA methylation analysis will be essential to define the full epigenomic consequences of transient MA exposure. Additionally, given emerging evidence that epigenetic modulation can enhance tumor immunogenicity and sensitivity to immune checkpoint blockade [49,50], combination strategies incorporating MA warrant exploration in the context of peritoneal disease.

While the present study identifies transcriptional modulation of several chromatin-associated regulatory factors, direct measurements of chromatin state were not performed. Future studies will be required to determine whether transient Mithramycin A exposure induces stable epigenetic alterations at the level of chromatin accessibility or DNA methylation.

## 4. Materials and Methods

### 4.1. Cell Lines and Culture Conditions

The human colorectal cancer cell lines HT-29 and Caco-2 were obtained from the American Type Culture Collection (ATCC, Manassas, VA, USA). Cells were authenticated by the supplier and used within 10 passages. Mycoplasma testing was performed routinely using PCR-based detection.

HT-29 cells were cultured in McCoy’s 5A medium supplemented with 10% fetal bovine serum (FBS) and 1% penicillin–streptomycin. Caco-2 cells were maintained in Dulbecco’s Modified Eagle Medium (DMEM) supplemented with 10% FBS and 1% penicillin–streptomycin. All cells were maintained at 37 °C in a humidified incubator with 5% CO_2_ unless otherwise specified.

### 4.2. Drug Treatments and HIPEC-Mimetic Exposure Conditions

Mithramycin A (MA), mitomycin C (MC), and oxaliplatin (OX) were purchased from Sigma-Aldrich (St. Louis, MO, USA) and prepared according to the manufacturer instructions.

To model HIPEC-relevant exposure conditions, HT-29 and Caco-2 cells were treated with either single agents or combination regimens consisting of 500 nM MA, 1.5 µg/mL MC, or 3.0 µg/mL OX for 90 min under normothermic (37 °C) or hyperthermic (42 °C) conditions.

For hyperthermic treatments, culture plates and drug-containing media were pre-equilibrated to 42 °C prior to application to ensure immediate and sustained thermal exposure throughout the 90-min incubation period. Hyperthermia was maintained using a temperature-controlled incubator calibrated to sustain stable 42 °C conditions.

Following the 90-min exposure, treatment media was removed and replaced with fresh drug-free medium for subsequent analyses.

### 4.3. RNA Extraction, RNA Sequencing, and Differential Expression Analysis

RNA sequencing was performed in biological triplicates for HT-29 cells under Control, Mithramycin A (MA), and Mitomycin C (MC) conditions. Library preparation and sequencing were conducted by Novogene (Beijing, China) using the Illumina NovaSeq 6000 platform with paired-end 150 bp reads, generating approximately 30 million read pairs (~60 million reads) per sample.

Raw sequencing reads were trimmed for adapter sequences using fastp [51]. Transcript-level quantification was performed using Salmon [52] against a transcriptome index derived from GENCODE v38 reference transcripts. Transcript-level estimates were summarized to gene-level counts using tximport [53] with the “scaledTPM” method.

Quality control assessment identified one MA-treated replicate (MA2) exhibiting high duplication rates and low mapping efficiency; this sample was excluded from downstream analyses according to standard RNA-seq quality filtering criteria. Analyses including and excluding MA2 yielded highly concordant differential expression and pathway enrichment results. Unless otherwise specified, results shown reflect analyses excluding MA2.

Low-abundance features were removed using the filterByExpr() function from edgeR [54]. Filtered counts were normalized using qsmooth normalization [55], and log_2_-qsmooth-normalized counts were used for differential expression analysis using the limma package with TREAT testing [56], applying fold-change threshold testing using the treat function [57].Limma has been widely applied to RNA-seq datasets when combined with appropriate normalization and filtering procedures and provides robust variance modeling, particularly in experiments with limited biological replicates [58].

Genes with |log_2_ fold change| ≥ 1.5 and adjusted *p*-value < 0.05 were considered significantly differentially expressed. Full differential expression results are provided in Supplementary Table S1. RNA sequencing data have been deposited in the Gene Expression Omnibus (GEO) under accession number GSE320366 and are currently under controlled access during the review process.

### 4.4. Gene Set Enrichment Analysis

Gene Set Enrichment Analysis (GSEA) was performed using the Broad Institute GSEA framework. Ranked gene lists were generated from differential expression output based on log_2_ fold change values.

Enrichment analysis was conducted using Hallmark gene sets from the Molecular Signatures Database (MSigDB), COSMIC cancer gene annotations, curated colorectal cancer tumor suppressor gene datasets, and Gene Ontology (GO) biological process categories. Normalized enrichment scores (NES) and false discovery rate (FDR) q-values were calculated using permutation-based testing. Pathways with FDR q < 0.05 were considered significantly enriched.

### 4.5. Cancer Gene Filtering Workflow

To identify clinically relevant MA-responsive genes, significantly differentially expressed genes (|log_2_FC| ≥ 1.5; adjusted *p* < 0.05) were cross-referenced with the COSMIC Cancer Gene Census, the Tumor Suppressor Gene Database (TSGD) specific for colorectal cancer.

Filtered genes were subsequently analyzed for enrichment in GO categories related to Cell-cycle regulation, Checkpoint signaling, and Negative regulation of proliferation.

This structured filtering approach prioritized mechanistically interpretable, cancer-relevant transcriptional changes.

### 4.6. Quantitative Real-Time PCR (qPCR)

Total RNA was isolated using the RNeasy Micro Kit (Qiagen, Hilden, Germany) according to the manufacturer’s instructions. RNA concentration and purity were assessed by spectrophotometry (NanoDrop 1000, Thermo Scientific, Waltham, MA, USA), and samples with A260/280 ratios of ~2.0 were used for downstream applications. cDNA was synthesized from 2 µg of total RNA using the High-Capacity cDNA Reverse Transcription Kit (Applied Biosystems, Foster City, CA, USA)) according to the manufacturer’s protocol.

Quantitative PCR was performed using SYBR Green Master Mix (Applied Biosystems, Foster City, CA, USA) on a QuantStudio real-time PCR system (Applied Biosystems, Foster City, CA, USA). Reactions were carried out in 20 µL volumes containing 4 ng of cDNA template and 200 nM of each primer. Cycling and melt curve analyses were performed according to manufacturer-recommended conditions.

Gene expression levels of DNMT1, JARID2, HDAC4, EZH2, CDKN1A, CDKN1C, CDKN2C, and CASP3 were normalized to GAPDH expression. Relative quantification was calculated using the 2^−ΔΔCt method. All reactions were performed in technical triplicate, and experiments were conducted independently at least three times.

### 4.7. Clonogenic Survival Assay

Following 90-min drug exposure and washout, cells were seeded at low density (500–1000 cells per well) in six-well plates and maintained under clonogenic conditions in drug-free growth medium for 14 days prior to colony fixation and quantification. Colonies were fixed with 4% paraformaldehyde and stained with 0.5% crystal violet. Colonies containing ≥50 cells were counted manually or using ImageJ software. (version 1.53, National Institutes of Health, Bethesda, MD, USA).

Clonogenic survival was calculated as follows:

Plating efficiency = (number of colonies formed/number of cells seeded) × 100

Relative survival was normalized to untreated controls.

### 4.8. Western Blot Analysis

HT-29 cells were treated with Mithramycin A (MA) for 90 min at either 37 °C or 42 °C, followed by drug washout and incubation in fresh medium. Forty-eight hours post-treatment, cells were washed with cold PBS and lysed in RIPA buffer supplemented with protease and phosphatase inhibitor cocktails. Lysates were incubated on ice for 1 h with intermittent vortexing and subsequently centrifuged at 15,000 rpm for 10 min at 4 °C. Supernatants were collected and stored at −80 °C until analysis.

Protein concentration was determined using the Pierce BCA Protein Assay Kit (Thermo Scientific, Rockford, IL, USA). Equal amounts of protein (20–30 µg) were heat-denatured and separated on 4–12% NuPAGE Bis-Tris gels (Thermo Scientific). Proteins were transferred to polyvinylidene difluoride (PVDF) membranes at 30 V for 90 min.

Membranes were blocked with 5% non-fat dry milk in TBST for 1 h at room temperature and incubated overnight at 4 °C with primary antibodies against cleaved caspase-3 (Cell Signaling Technology, Danvers, MA, USA; catalog no. 9661S; dilution 1:1000), PARP (Cell Signaling Technology; catalog no. 9542; dilution 1:1000), and β-actin (Cell Signaling Technology; clone 13E5; catalog no. 4970; dilution 1:2000). After washing, membranes were incubated with HRP-linked anti-rabbit IgG secondary antibody (Cell Signaling Technology; catalog no. 7074; dilution 1:5000) for 1 h at room temperature.

Protein bands were detected using enhanced chemiluminescence (Pierce™ ECL Western Blotting Substrate; Thermo Scientific) and imaged using the iBlot imaging system (Thermo Fisher Scientific). Band intensities were quantified using ImageJ software (NIH), and the apoptotic index was calculated as the ratio of cleaved PARP to full-length PARP normalized to β-actin.

### 4.9. Statistical Analysis

All experiments were performed using at least three independent biological replicates unless otherwise specified. Data are presented as mean ± standard deviation (SD). Statistical analyses were conducted using GraphPad Prism (version 10.6.1, GraphPad Software, San Diego, CA, USA).

Comparisons between two groups were performed using unpaired two-tailed Student’s *t*-tests. For experiments involving two independent variables (Mithramycin A concentration and temperature), two-way ANOVA was performed. Where appropriate, post hoc comparisons were conducted using Šidák’s multiple comparisons test to evaluate differences between temperatures at each concentration level. A *p*-value < 0.05 was considered statistically significant.

AI Disclosure Statement: The authors used a generative artificial intelligence tool to assist with language refinement and manuscript structuring. All scientific content, experimental design, data analysis, interpretation, and conclusions were developed and verified by the authors, who take full responsibility for the integrity and accuracy of the work.

## 5. Conclusions

Short-duration HIPEC-mimetic exposure to Mithramycin A induces coordinated transcriptional and epigenetic reprogramming in colorectal cancer models. These effects suggest a complementary therapeutic mechanism beyond conventional DNA-damaging HIPEC agents, involving modulation of oncogenic transcriptional programs and checkpoint-associated signaling. Our findings support the potential of transient exposure to transcriptionally active compounds as part of locoregional treatment strategies. Future studies should validate these effects in vivo and further define the epigenomic consequences of transient Mithramycin A exposure, including chromatin accessibility and DNA methylation dynamics.

## Data Availability

The RNA sequencing data generated during the current study have been deposited in the NCBI Gene Expression Omnibus (GEO) under accession number GSE320366. All additional data are available within the article and its Supplementary Materials.

## Supplementary Materials

The following supporting information can be downloaded at: https://www.mdpi.com/article/10.3390/ijms27083580/s1.
