# Supplementary material for: Short-Duration HIPEC-Mimetic Mithramycin A Exposure Induces Durable Transcriptional Remodeling Involving Chromatin Regulatory Networks in Colorectal Cancer Models
Source: Int J Mol Sci. 2026 Apr 17;27(8):3580. doi: 10.3390/ijms27083580 (PMC13116636; doi:10.3390/ijms27083580)
Supplement: Supplementary file 1 [file ijms-27-03580-s001.zip › Supplementary Figure S2 GO enrichment (ribosome, small GTPase, etc.).pdf]

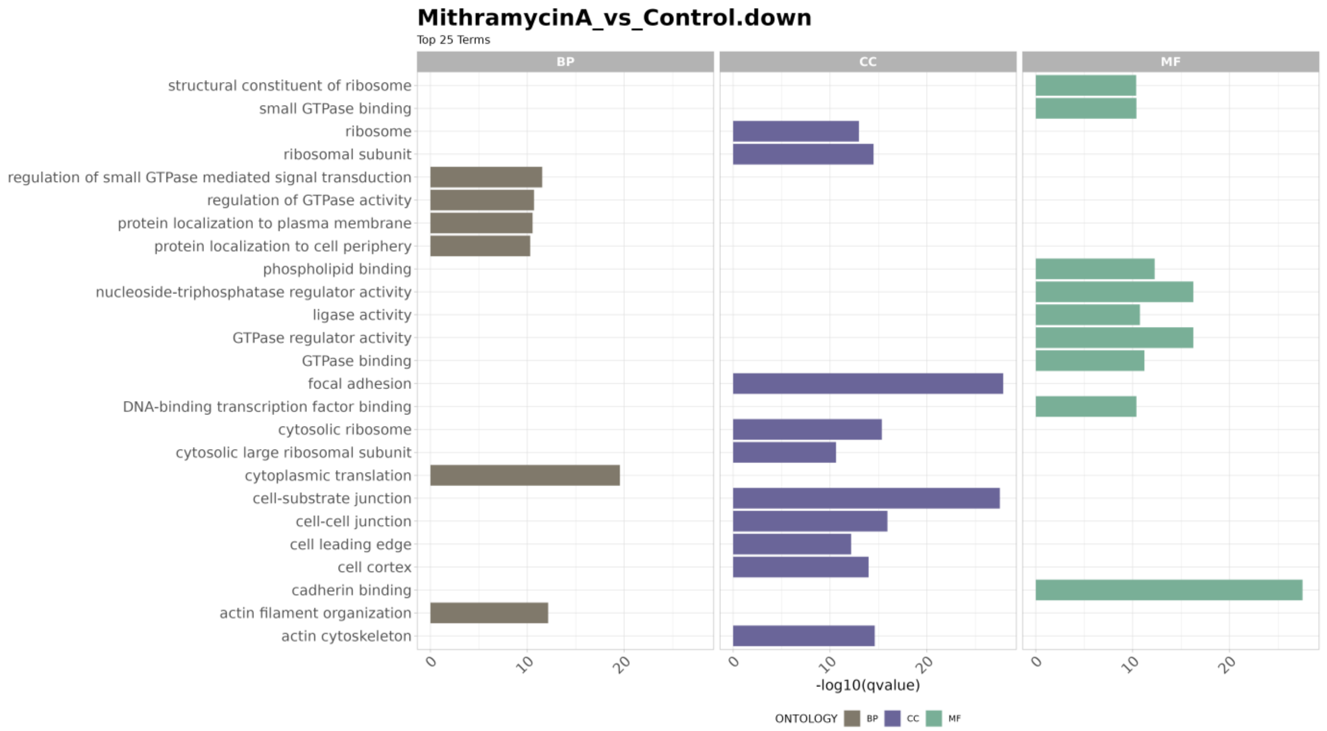


- Ribosome
- Small GTPase
- Cell junction
- DNA-binding

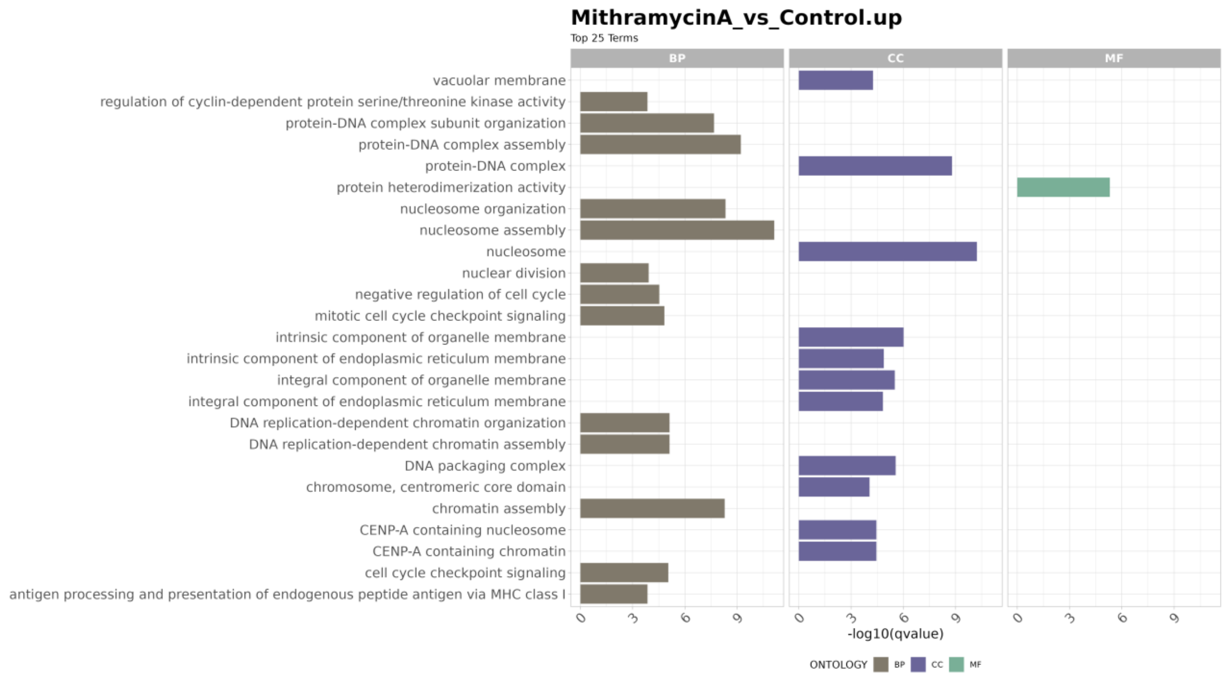


- DNA, chromatin
- Cell cycle

BP: Biological Process
MF: Molecular Function
CC: Cellular Component.
