# Supplementary material for: Short-Duration HIPEC-Mimetic Mithramycin A Exposure Induces Durable Transcriptional Remodeling Involving Chromatin Regulatory Networks in Colorectal Cancer Models
Source: Int J Mol Sci. 2026 Apr 17;27(8):3580. doi: 10.3390/ijms27083580 (PMC13116636; doi:10.3390/ijms27083580)
Supplement: Supplementary file 1 [file ijms-27-03580-s001.zip › Supplementary Figure S4 PCA.pdf]

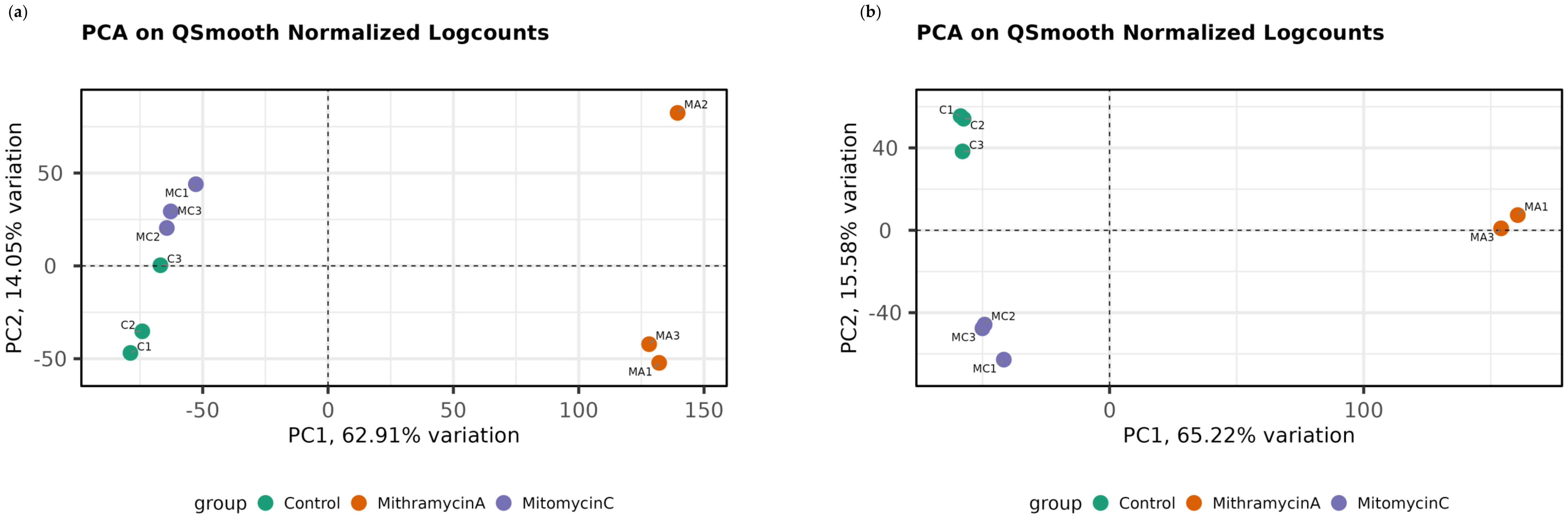
(a)
PCA on QSmooth Normalized Logcounts
PC2, 14.05% variation
PC1, 62.91% variation
50
0
-50
-50
0
50
100
150
MA2
MC1
MC3
MC2
C3
C2
C1
MA3
MA1
group
Control
MithramycinA
MitomycinC
(b)
PCA on QSmooth Normalized Logcounts
PC2, 15.58% variation
PC1, 65.22% variation
40
0
-40
0
100
C1
C2
C3
MA1
MA3
MC2
MC3
MC1
group
Control
MithramycinA
MitomycinC
