# Supplementary figures and images for: Short-Duration HIPEC-Mimetic Mithramycin A Exposure Induces Durable Transcriptional Remodeling Involving Chromatin Regulatory Networks in Colorectal Cancer Models
Source: Int J Mol Sci. 2026 Apr 17;27(8):3580. doi: 10.3390/ijms27083580 (PMC13116636; doi:10.3390/ijms27083580)

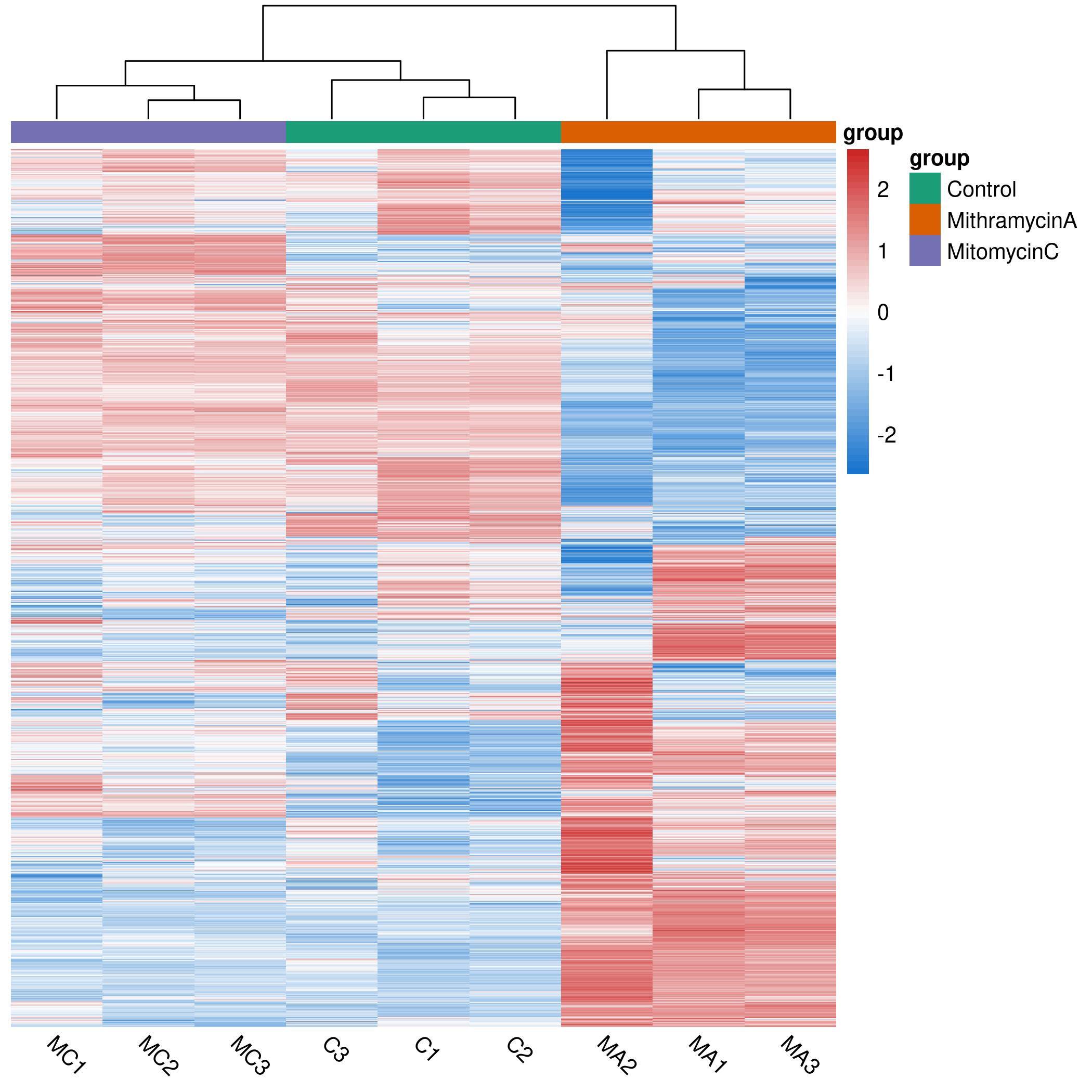

Supplement: Supplementary file 1 [file ijms-27-03580-s001.zip › Supplementary Figure S1 heatmap.png]

(a)

GSEA Results; All Contrasts, All Pathways

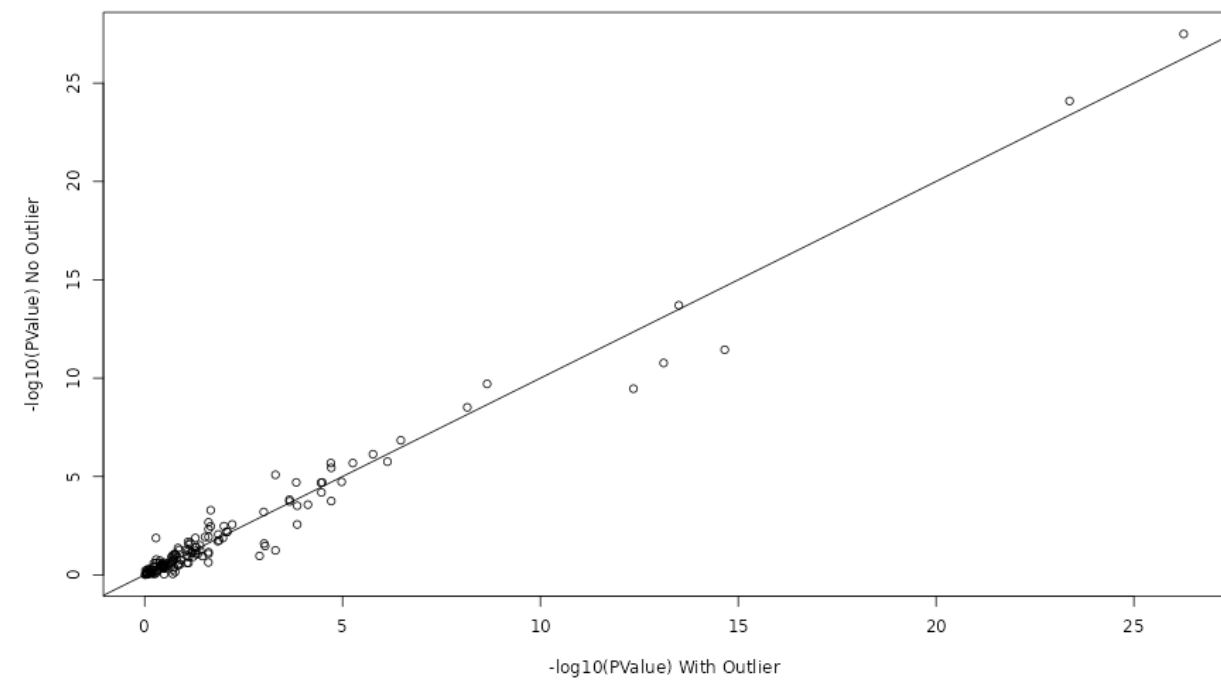

(b)

GSEA Results; All Contrasts, All Pathways

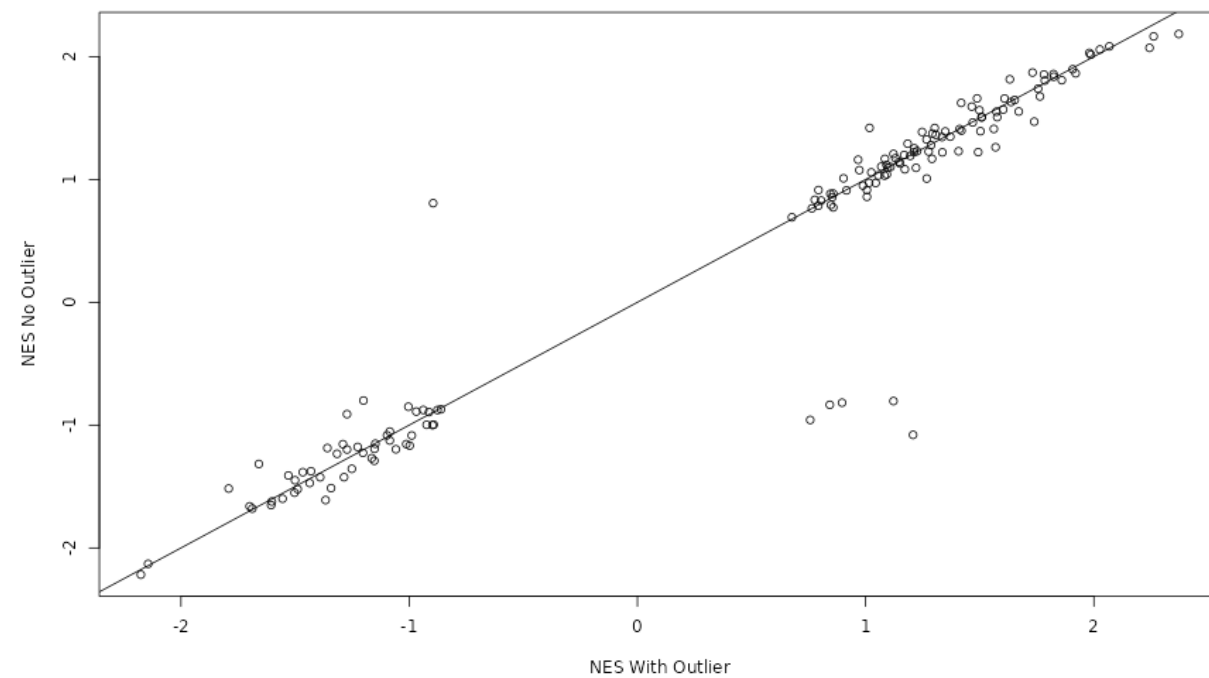

Supplement: Supplementary file 1 [file ijms-27-03580-s001.zip › Supplementary Figure S3. Correlation of Gene Set Enrichment Analysis (GSEA).pdf]

(a) General Statistics

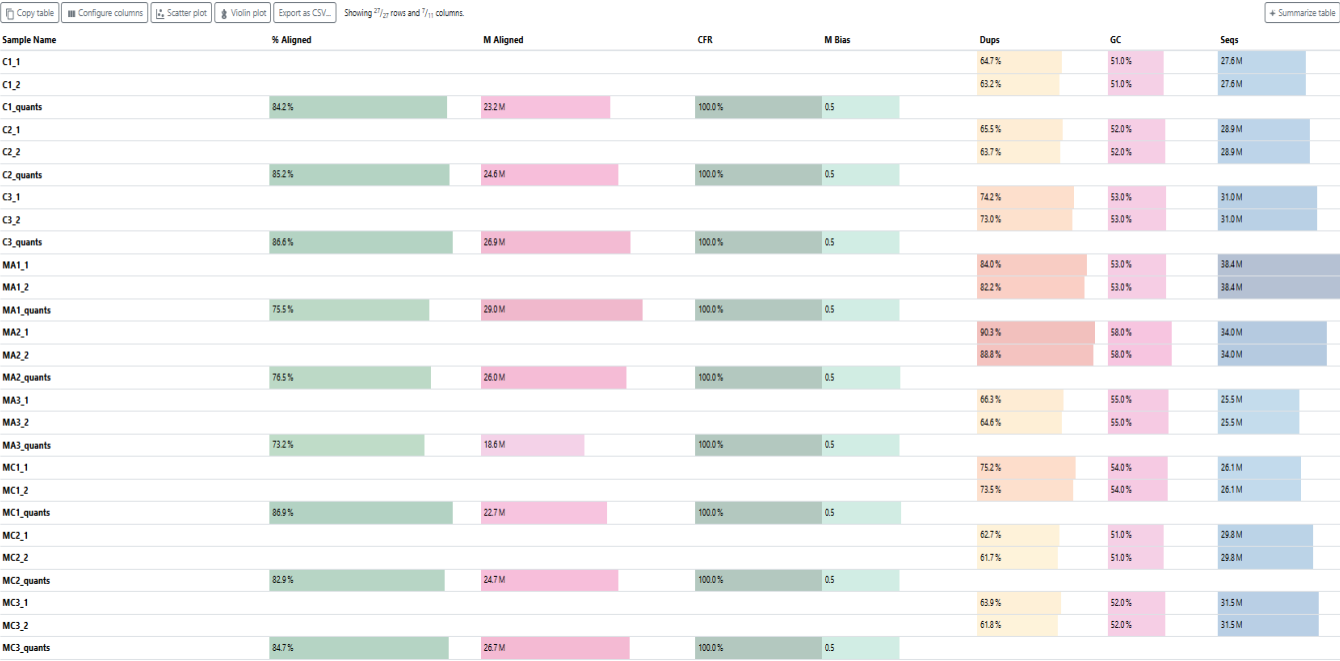

(c)

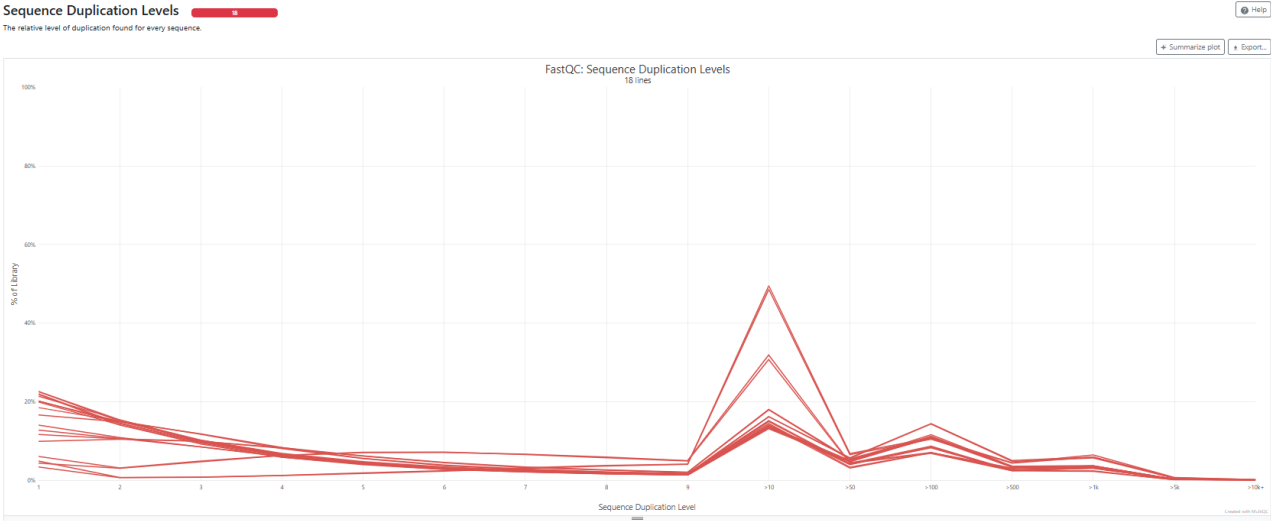

(b)

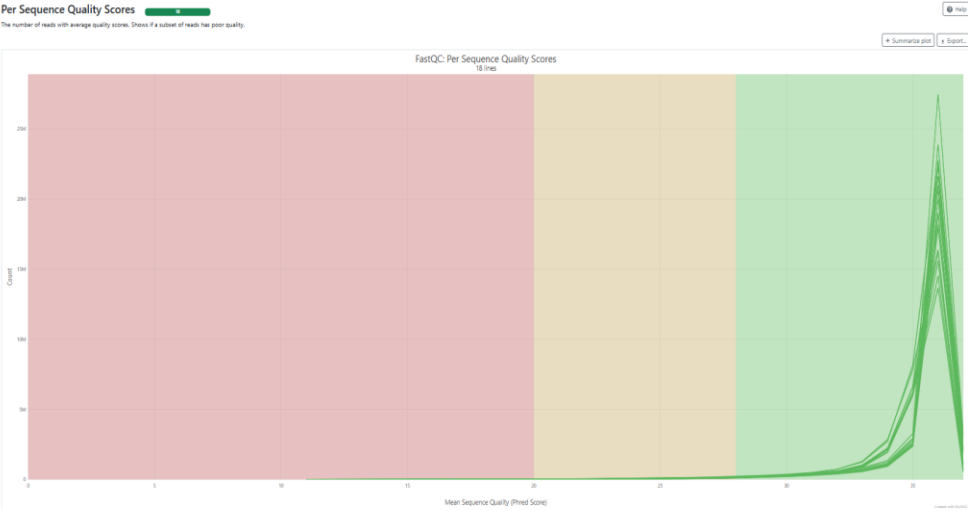

(d)

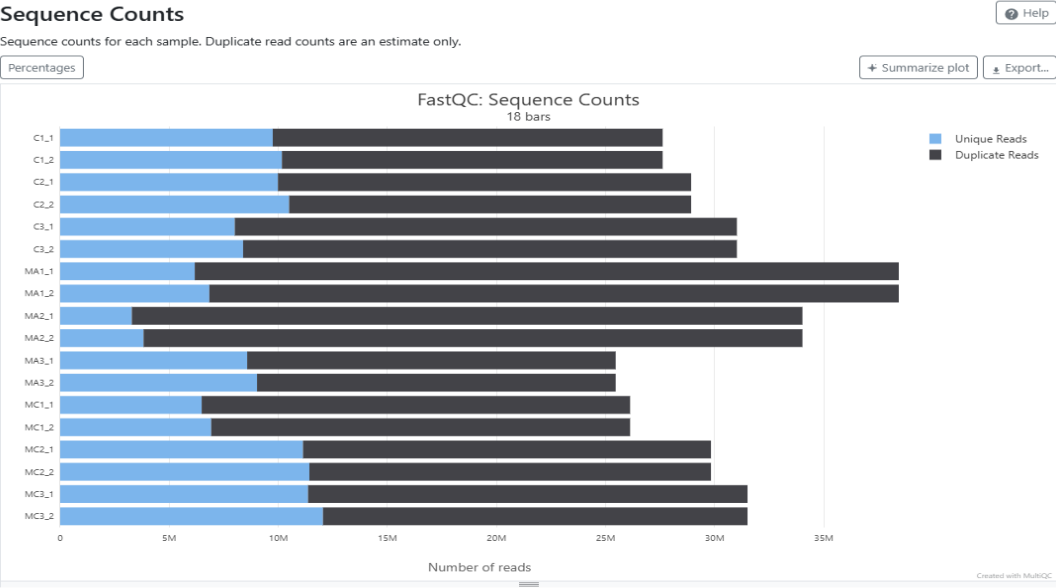

Supplement: Supplementary file 1 [file ijms-27-03580-s001.zip › Supplementary Figure S5 RNA-seq quality control metrics.pdf]
